# Follicular Transplantation, Microneedling, and Adjuvant Narrow-band Ultraviolet-B Irradiation as Cost-Effective Regimens for Palmar-Plantar Vitiligo: A Pilot Study

**DOI:** 10.7759/cureus.7878

**Published:** 2020-04-28

**Authors:** Amir Feily, Abdollah Firoozifard, Toktam Sokhandani, Perla Elosegui-Rodriguez, Evian Perez-Rivera, Christopher S Lange, Masoomeh Hosseinpoor, Marigdalia K Ramirez-Fort

**Affiliations:** 1 Dermatology, Jahrom University of Medical Sciences, Jahrom, IRN; 2 Pathology, San Juan Bautista School of Medicine, Caguas, PRI; 3 Radiation Oncology, State University of New York Downstate Health Sciences University, Brooklyn, USA; 4 Otolaryngology, Mashhad University of Medical Sciences, Mashhad, IRN; 5 Life Sciences, Biofort Corp., Guaynabo, PRI

**Keywords:** vitiligo, micro needling, nbuvb, melanocytic stem cells, hair transplantation, co2 fractional laser, ultraviolet radiation (uvr) therapy

## Abstract

Treatment of refractory palmar-plantar vitiligo is particularly challenging because the skin in these regions has a limited supply of follicle-derived melanocytic stem cells. Autologous hair transplantation monotherapy is effective in some forms of vitiligo through the provision of melanocytic stem cells. CO_2_ laser followed by exposure to light (i.e., sunlight or narrow-band ultraviolet-B [nbUVB]) has independently shown to be an effective treatment strategy. Recently, it was found that the combination of hair transplantation and CO_2_ laser followed by nbUVB exposure had superior efficacy to either modality as monotherapy. Similar to CO_2_ laser, microneedling produces skin cell proliferation and releases pro-pigmentary cytokines. Given the important role of the cytokines in vitiliginous skin, microneedling may also be an effective therapeutic modality for refractory vitiligo. Herein, we conducted a pilot study to evaluate the efficacy of hair transplantation and CO_2_ laser or microneedling followed by nbUVB. Microneedling and fractional CO_2_ laser in combination with hair transplantation and nbUVB both demonstrated utility in the induction of repigmentation in refractory palmar-plantar vitiligo; however, a larger trial would be needed to determine a difference in treatment efficacy. Nonetheless, microneedling is cost-effective and requires minimal training; therefore, microneedling can be easily incorporated into standard dermatological practice.

## Introduction

Treatment of stable and refractory palmar-plantar vitiligo is particularly challenging because the skin in these regions is inherently void of hair follicles. Follicles are an important source of melanocytic stem cells needed for repigmentation [1-3]. Therefore, hair follicle transplantation, while time-consuming, is a valuable mechanism to introduce pigmentary stem cells into the palmar-plantar regions [4-7]. An increasing amount of literature underlines the efficacy of hair follicle transplantation as a valid monotherapy for cutaneous repigmentation [4-7]. Currently, hair follicle transplantation is being used in different therapeutic protocols, which combine fractionated CO_2_ laser or microneedling with narrow-band ultraviolet-B (nbUVB) phototherapy and topical steroids [8-12]. Both fractional CO_2_ laser and microneedling as therapeutic options for vitiligo seem to be independently useful, but there are no comparative studies to date.

In the recent past, our group described the validity and efficacy of a quadrimodal therapy with hair transplantation, fractionated CO_2_ laser, topical steroids, and nbUVB for refractory vitiligo [9]. The improved efficacy of the quadrimodal treatment occurred by the presumed mechanisms of melanotoxin depletion, increased topical steroid delivery through ablative channels, and induced upregulation of pro-pigmentary cytokines within the lesional microenvironment; collectively, these changes combined with nbUVB stimulation are thought to enhance stem cell viability and melanin production [8-12]. Although nbUVB can be quite effective as monotherapy, the lack of hair follicles (and source of melanocytic stem cells) in palmar-plantar vitiliginous skin limits its use in this vitiligo phenotype.

Similar to fractionated CO_2_ laser, microneedling is a therapeutic modality that produces skin cell proliferation and releases pro-pigmentary cytokines [12,13]. Specifically, it accomplishes this by inserting needles onto the skin and promoting healing [14]. Given the important role of cytokines in vitiliginous skin, microneedling may be a cost-effective therapeutic modality (as compared to fractional CO_2 _laser) for refractory vitiligo [8,9,12,13,15]. The traumatic inflammatory infiltrate from microneedle punctures may optimize regional antigen presentation and wound healing, leading to the removal of pathogenic cells and melanotoxins.

On the other hand, fractional CO_2_ laser is a tissue-selective treatment used for many dermatological diseases that emits light energy and fractionates it into microbeams that are delivered in a certain number of sessions, or fractions, to the tissue [16]. The microbeams are not pigment-selective and are absorbed by water, mostly found in soft tissues. The energy is delivered at high peaks and short duration to induce inflammation in the intended tissue and minimizes damage of the normal surrounding tissue. The devices that emit the fractional CO_2_ laser cost approximately $25,000, whereas the microneedling devices cost approximately $1,000, but prices vary by vendor. Compared with fractional CO2 laser, the cost of the microneedling procedure is significantly less to the practicing dermatologist; therefore, microneedling quadrimodality (i.e., microneedling, hair transplantation, topical steroids, nbUVB) is a protocol that could be conveniently incorporated into standard practice for the therapeutic management of refractory palmar-plantar vitiligo.

IIn this study, we aim to compare the efficacy of microneedling with fractionated CO_2_ laser, as part of a multimodal regimen including hair follicle transplantation, nbUVB, and topical clobetasol solution, in the treatment of stable and refractory palmar-plantar vitiligo.

## Materials and methods

A prospective pilot study was conducted from January 2015 to January 2016. Twenty patients (10 women; 10 men) with Fitzpatrick skin type IV were enrolled in the study; two male patients withdrew consent prior to starting treatment. All patients were diagnosed with stable and refractory palmar-plantar vitiligo by board-certified dermatologists. Stable and refractory vitiligo was defined as lesions that did not progress over a 12-month timeframe and that had not previously responded to standard monotherapies or combined ones (e.g. topical steroids, nbUVB) (Table 1). Major exclusionary criteria included (1) hypersensitivity to laser and hair transplantation materials, (2) a history of photosensitivity, (3) a personal history of keloid formation, and (4) a personal history of Koebnerization.

**Table 1:** Prior failed treatment modalities in enrolled patients. Nb, narrow-band; UVB, ultraviolet B rays

Treatment	Duration	Number of patients
Topical corticosteroids	6 weeks	15
Topical calcineurin inhibitors	2 months	5
Nb-UVB	15 weeks	20
Nb-UVB + topical corticosteroids	15 weeks	5

Comparable vitiliginous lesions were selected from both sides of the body. On day 0, heavily pigmented follicular grafts were harvested from the scalp by follicular unit extraction and transplanted in a 1-cm grid pattern throughout the selected lesions. At days 30±4 and 60±4, left-sided lesions received single fractions with a fractional CO2 laser, MX-7000 (10,600 nm, 100-MJ pulse energy, and 200 spots/cm3 in static mode) (Daeshin Enterprise Corporation, IDS CO, Seoul, Korea), under topical anesthesia with lidocaine-prilocaine cream applied for 20 minutes prior to intervention. Right-sided lesions received 1.5- to 2-mm needle length-assisted microneedling until pinpoint bleeding was achieved. Topical anesthesia with lidocaine-prilocaine cream was used for 20 minutes prior to microneedling. On days 30±4 and 60±4 after primary investigational treatment (i.e., fractional CO2 laser or microneedling), silver sulfadiazine ointment was applied to the treated skin, twice daily, for five days without occlusion.

On day 41±2, right- and left-sided lesional skin received nbUVB phototherapy (Dermalight 1,000/1,000 L, 800 W; power supply: 230 V/50 Hz; Dr. Honle Medizinitechnik GmbH, Gilching, Germany) three times a week until day 60±4; phototherapy was restarted on day 71±2. The phototherapeutic dose was increased by 15% at each treatment interval and was completed by day 100±4 (for a total of 12 weeks of treatment). Clobetasol 45% solution (45% clobetasol in 100-mL isopropyl alcohol) was applied twice daily throughout the phototherapy phase of the study.

The diameter of repigmentation around each graft was photographed and measured with appropriately calibrated calipers at baseline: days 30±4, 60±4, and 100±4. Treatment toxicities were evaluated at each patient visit and measured according to the Common Terminology Criteria for Adverse Events (CTCAE v4.0). On day 100±4, patients completed a treatment satisfaction questionnaire (scale of 0 to 10, unsatisfied to highly satisfied).

Data were analyzed by the SPSS software (IBM Corp., Armonk, NY, USA) using the Mann-Whitney and Friedman tests.

## Results

Twenty patients (10 women; 10 men) with Fitzpatrick skin type IV were enrolled in the study; two male patients withdrew consent prior to starting treatment. The mean patient age was 30.22 years. The mean duration of disease ±SD was 10.4±5.8 years. There was an even distribution (i.e., 1:1 ratio) of palmar and plantar lesions treated.

**Figure 1:**
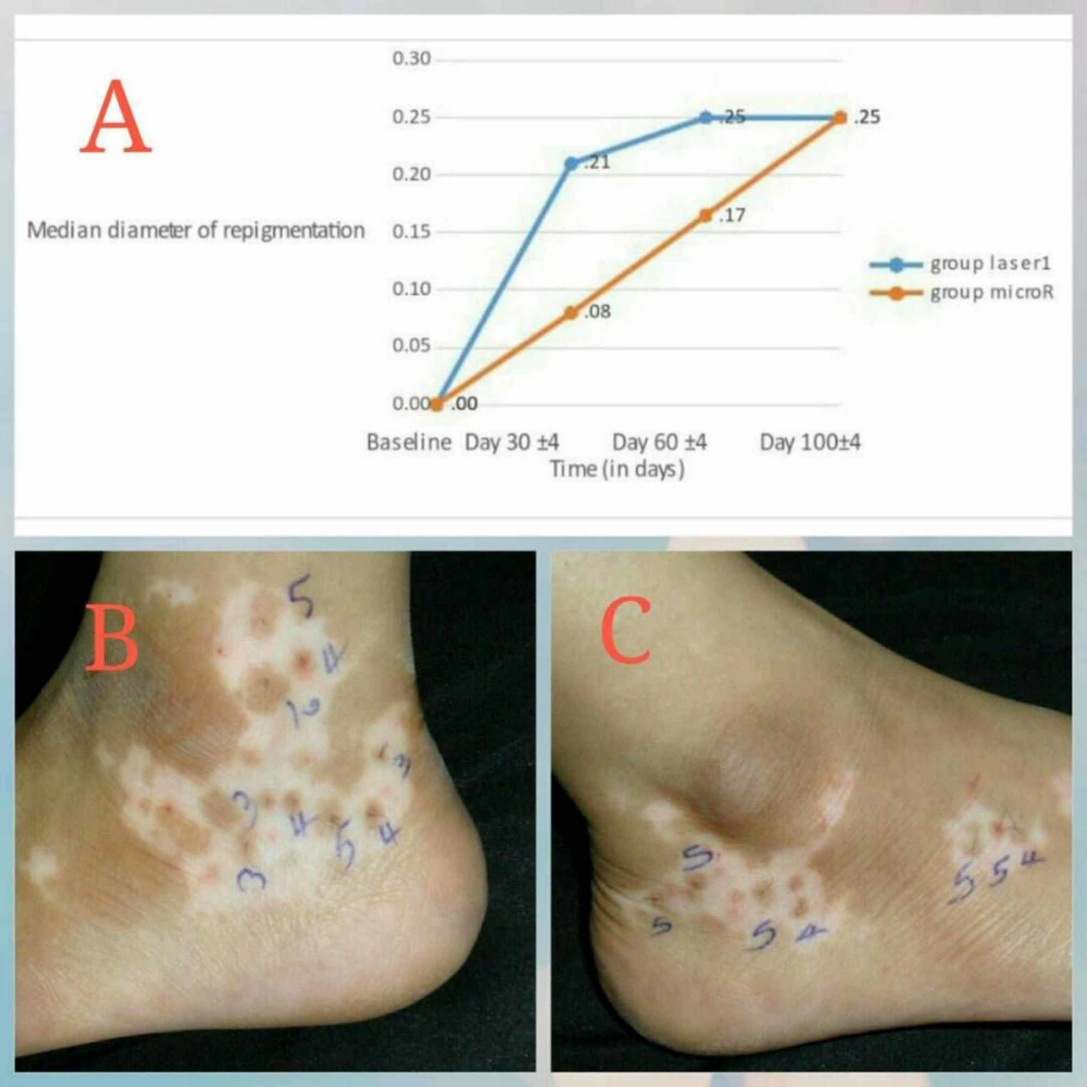
Rate of repigmentation by treatment arms. (A) Graphically expressed diameter of repigmentation around each graft. (B and C) Photographed repigmentation.

A total of 95% of transplanted follicles survived; all remaining follicles maintained pigment throughout the study. At days 30±4 and 60±4, there was 0.21 mm and 0.25 mm of repigmentation after hypofractionated CO_2_ laser compared with 0.08 mm and 0.17 mm in lesions treated with microneedling (p=0.74 and p=0.86 for days 30±4 and 60±4, respectively) (Figure 1A-C) (Table 2). By day 100±4, 78% of grafts demonstrated repigmentation, which measured 0.25 mm after hypofractionated CO_2_ laser and 0.25 mm after microneedling (p= 0.84). By power analysis, 93 patients are needed to have an 80% chance of not rejecting a true difference in efficacy between hypofractionated CO_2_ laser and microneedling.

**Table 2:** Diameter of repigmentation by treatment arms.

Follow-up time	Treatment arms		p-Value
	Laser (mm), median (range)	Microneedling (mm), median (range)	
Baseline	0.00 (0-0)	0.00 (0-0)	-
Day 30±4	0.21 (0-2)	0.08 (0-1)	0.74
Day 60±4	0.25 (0-2.57)	0.17 (0-1.30)	0.86
Day 100±4	0.25 (0-3)	0.25 (0-1.66)	0.84

All patients experienced grade 1 erythema and grade 1 pain in both treatment arms after fractional CO_2_ laser and microneedling. Grades 2 to 5 toxicities were not identified. High patient satisfaction was noted in both treatment groups: a treatment satisfaction questionnaire (i.e., patient-reported outcome forms) did not identify a difference in patient preference toward one of the two modalities.

## Discussion

Our pilot study indicates that quadrimodal therapy with microneedling or hypofractionated CO_2_ laser is efficacious in stable and refractory palmar-plantar vitiligo. The 78% overall response rate achieved by our patients supports that both microneedling and hypofractionated CO_2_ laser have similar efficacies in improving pigmentation of vitiliginous lesions when follicular transplantation, topical steroids, and nbUVB are incorporated [1,17]. Furthermore, both modalities result in comparable patient satisfaction.

The initial slope of the hypofractionated CO_2_ laser repigmentation curve was steeper when compared with microneedling, but the difference was not significant (Figure 1). The faster rate of repigmentation might be explained by the added benefit of the ablative channels created by the CO_2_ laser. Our pilot study was not powered to detect non-inferiority. A larger trial of at least 93 patients is needed to evaluate non-inferiority of either modality, but costs versus benefits support the use of microneedling in practices that do not have readily accessible fractional CO_2_ laser capabilities.

Prior studies have evaluated the efficacy of fractional CO_2_ laser (without transplantation of melanocytic stem cells) in combination with phototherapy (e.g., sunlight or targeted UVB) [10,18]. The results of these studies are contradictory. Fractional CO_2_ laser followed by sunlight (i.e., wide range of UV wavelengths) exposure resulted in acceptable repigmentation of refractory vitiligo, with a repigmentation rate ranging from 24% to 74% [10]. However, fractional CO_2_ laser followed by targeted UVB with topical steroids had no improvement in pigmentation compared with targeted UVB and topical steroids alone [18]. The latter findings suggest that the addition of CO_2_ laser treatment alone does not improve the efficacy of UV light with topical steroids. CO_2_ laser hypofractionation and transplantation of viable melanocytic stem cells is required to improve repigmentation [9]. We optimized the propigmentary environment by hypofractionating the CO_2_ laser sessions from 10 sessions to 2 sessions to prevent ablation of viable, residing melanocytes [9,18]. We also implanted richly pigmented hair follicles as a source of healthy melanocytic stem cells [9]. CO_2_ laser hypofractionation should theoretically minimize the risk of vitiliginous Koebnerization, thereby leading to better clinical results [9,18].

Stanimirovic et al. compared latanoprost in combination with nbUVB phototherapy with latanoprost plus one session of 0.5-mm needle length-assisted skin microneedling in combination with nbUVB phototherapy to induce repigmentation in refractory vitiligo lesions [19]. The results showed that latanoprost with nbUVB was effective, but microneedling had no improvement upon this efficacy. The effectiveness of microneedling observed in our study may be explained by differences in session numbers, technique, and medical equipment. The performance of more microneedling sessions with longer needle lengths (i.e., Dermaroller® versus Dermapen®) should induce a larger inflammatory infiltrate and propigmentary cytokine release.

Hair follicle transplantation was standard to both arms of the study. A potential adverse outcome with the use of melanocytic stem cells from hair follicles is the poor cosmesis of new hairs on the palmar and plantar skin. Therefore, patient education should be provided prior to the hair follicle transplantation procedure. In our experience, five patients showed hair growth in the treated areas, which was resolved with laser hair removal performed after complete patch repigmentation.

## Conclusions

Microneedling or fractional CO_2_ laser in combination with hair transplantation, topical steroids, and nbUVB are utile and effective in the induction of repigmentation in stable and refractory palmar-plantar vitiligo. The initial rate of repigmentation with the hypofractionated doses of fractional CO_2_ laser was faster than with microneedling, but this difference was not significant with the sample size of our pilot study. A larger trial would be needed to determine a difference in treatment efficacy. However, microneedling is more cost-effective and requires minimal training; therefore, microneedling can be easily and efficiently incorporated into standard dermatological practice.
